# Epidemiological Assessment of Benzodiazepine Dependence via Pharmacist-Led EMR Review in Pain and Palliative Care Institution

**DOI:** 10.3390/pharmacy14010006

**Published:** 2026-01-07

**Authors:** Carlos Eduardo Estrada-De La Rosa, Felipe Alexis Avalos-Salgado, Daniel Osmar Suárez-Rico, Martin Zermeño-Ruiz, César Ricardo Cortez-Álvarez, Raymundo Escutia-Gutiérrez

**Affiliations:** 1 Instituto Jalisciense de Alivio al Dolor y Cuidados Paliativos, Secretaria de Salud Jalisco, Zapopan 45150, Mexico; carlos.estrada@red.jalisco.gob.mx; 2 Departamento de Farmacobiología, Centro Universitario de Ciencias Exactas e Ingenierías (CUCEI), Universidad de Guadalajara, Guadalajara 44430, Mexico; alexis.avalos@academicos.udg.mx (F.A.A.-S.); daniel.suarez@academicos.udg.mx (D.O.S.-R.); martin.zermeno@academicos.udg.mx (M.Z.-R.); cesar.cortez@academicos.udg.mx (C.R.C.-Á.); 3 Instituto de Terapéutica Experimental y Clínica (INTEC), Centro Universitario de Ciencias de la Salud (CUCS), Universidad de Guadalajara, Guadalajara 44340, Mexico

**Keywords:** benzodiazepines, drug dependence, BDEPQ-MX, pharmacist, palliative-care, chronic pain, risk factors

## Abstract

Background/Objectives: Benzodiazepines (BZDs) are used routinely in cases requiring sedation for anxiety, insomnia, and procedures that require pain management, and daily use of these agents may extend over several months; therefore, monitoring patients is essential to reduce the risk of developing dependence. However, the high patient volume in pain and palliative-care settings often limits physicians’ ability to both conduct consultations and perform comprehensive evaluations. In this context, the pharmacist plays a key role in supporting patient care by contributing professional activities that enhance patient well-being, such as conducting systematic reviews of electronic medical records. This pharmacist-led EMR assessment enables the identification of benzodiazepine dependence patterns and supports a more robust epidemiological evaluation within the institution. Methods: A descriptive observational study (January 2022–May 2025) using electronic medical records and prescription data was conducted. Consecutive adults with an active BZD prescription and a documented BDEPQ-MX (Benzodiazepine Dependence Questionnaire, Mexican version) were included. Outcomes were BDEPQ-MX categories (No dependence; Pleasurable effects; Perceived need; Dependence) and a binary endpoint was stablished as “any dependence” (either scored in Perceived need or Dependence category) vs. No dependence (either scored as No dependence or Pleasurable effects categories). Group comparisons used χ^2^, Student’s t, and one-way ANOVA. A logistic regression modeled any dependence; a general linear model (GLM) examined the BDEPQ-MX total score. Results: Of 181 complete cases, BDEPQ-MX categories were No dependence 33.2% (60/181), Pleasurable effects 7.2% (13/181), Perceived need 17.1% (31/181), and Dependence 42.5% (77/181); hence, 59.7% met “any dependence.” Women comprised 67.4% overall. Compared with No dependence, the any-dependence group had higher comorbidity (83.3% vs. 65.8%, *p* = 0.006) and markedly greater duration of BZD use (months) (22.6 ± 11.5 vs. 5.9 ± 4.9, *p* < 0.001), with no difference in daily dose (*p* = 0.6). Benzodiazepine medications shifted toward alprazolam in dependence (38.9% vs. 20.5%, *p* = 0.009) and away from clonazepam (43.5% vs. 58.9%, *p* = 0.042). In the adjusted model, the male sex was associated with lower odds of any dependence (aOR 0.29, 95% CI 0.11–0.76; *p* = 0.013), while the duration of BZD use (per month) increased the odds (aOR 1.32, 1.20–1.45; *p* < 0.001). In the GLM, the duration showed the largest effect on BDEPQ-MX total (F = 203.26; *p* < 0.001; partial η^2^ = 0.545). Conclusions: In this outpatient pain and palliative-care population, benzodiazepine-related dependence phenomena were common: 59.7% of patients met the criteria for dependence based on the pharmacist-led EMR review. The involvement of the pharmacist was essential, as this systematic evaluation would have been difficult to perform within routine medical consultations. The pharmacist’s contribution enabled a detailed epidemiological characterization, revealing that the exposure duration—more than daily dose—was the principal, modifiable correlate of dependence, and that alprazolam was disproportionately represented in the higher-dependence categories. These findings underscore the value of pharmacist-supported surveillance to identify and measure BZD dependance.

## 1. Introduction

Benzodiazepines (BZDs) remain widely prescribed for anxiety and insomnia, yet long-term use is common despite guidance favoring short courses. Prolonged exposure is associated with daytime sedation, psychomotor slowing, cognitive impairment, falls, and clinically significant dependence—especially in older adults and women [1,2]. Real-world studies consistently show higher use in females and late-life patients, frequent chronic use beyond recommended durations, and a preference for a small subset of agents (e.g., alprazolam, lorazepam, clonazepam) [3,4]. In Latin America, population studies similarly document sustained BZD and Z-agent consumption with demographic gradients that align with international trends [5].

Patient perceptions and routine practice patterns help explain the persistence. Qualitative syntheses report that many long-term users view BZDs as essential for daily functioning, minimize or feel resigned to long-term risks, and anticipate physician-led guidance before reducing or stopping; psychological dependence and limited support for tapering further entrench continued use [6,7]. In outpatient samples, self-identified “dependence” often reflects the inability to down-titrate without discomfort, which users distinguish from “addiction/abuse,” complicating risk communication and deprescribing efforts [8]. At the system level, prescribing and dispensing studies highlight gaps in initiation/review processes (e.g., suboptimal documentation of indication and duration, limited planned discontinuation), which correlate with longer exposure and higher dependence risk [9,10].

Accurate measurement of BZD-related dependence phenomena is therefore central to clinical care and epidemiology. The Benzodiazepine Dependence Questionnaire (BDEPQ) was developed to quantify dependence on a continuum across three core dimensions—Perceived need, Pleasant effects, and general Dependence—complementing (but not replacing) the assessment of acute withdrawal [11]. In Mexico, the BDEPQ-MX (Mexican adaptation/validation of the Benzodiazepine Dependence Questionnaire) demonstrated a stable three-factor structure, excellent internal consistency, and strong diagnostic performance versus SCID-I, supporting a pragmatic cut-off for clinical screening in psychiatric outpatients [12]. However, data on BZD dependence in pain relief and palliative-care services—where anxiety, insomnia, and procedural sedation needs are common and daily BZD use may extend over months—remain sparse [13,14,15].

In pain and palliative-care settings, clinicians frequently work under significant time pressure, which limits their ability to conduct the comprehensive monitoring required for safe benzodiazepine use. Evidence shows that many physicians report needing more time than allotted to properly evaluate their patients, and that shortened consultations compromise the detection of treatment-related risks [16,17]. The objective of this study was to assess BZD dependence via an EMR review, which would allow us to demonstrate that, beyond the medical consultation, it is possible to identify and estimate the prevalence of different degrees of benzodiazepine dependence through a systematized review of validated questionnaires applied during medication dispensing. This approach would enable institutions to design strategies to support their patients and to train healthcare personnel accordingly.

## 2. Materials and Methods

We performed a descriptive observational study of outpatients seen at the Jalisco Institute for Pain Relief and Palliative Care (Zapopan, Mexico) between January 2022 and May 2025. The institute is a state public health facility providing multidisciplinary care for chronic pain and palliative-care patients.

Database elaboration: The questionnaire BDEPQ-MX was administered during BZD agents dispensing by the pharmacists in charge of the service to patients; the indications for which benzodiazepines were prescribed are listed as follows: insomnia, anxiety disorder, affective disorder, mixed anxiety–depressive disorder, and major depressive disorder. No patients in a palliative-care setting requiring these medications were identified at our center. The data were subsequently entered into the patient’s electronic medical record, the “Health Record System” (Sistema de Registro en Salud, SRS). Through a subsequent systematized review of patients with a history of benzodiazepine prescription, we screened consecutive outpatients with an active benzodiazepine (BZD) prescription documented in the electronic medical record (EMR) during the study window.

Inclusion criteria: (i) age ≥ 18 years; (ii) at least one follow-up visit during which the BDEPQ-MX was administered; (iii) available data on BZD drug and daily dose.

Exclusion criteria: (i) missing BDEPQ-MX total score; (ii) incomplete key covariates (sex or age); (iii) documented severe cognitive impairment precluding valid questionnaire completion. Of the screened patients, *n* = 181 met criteria and formed the analytic sample. For these patients, an Excel database was created using the information collected from the aforementioned electronic and manual records, which was then used for the corresponding analysis.

The BDEPQ-MX was selected because it was validated in Mexican patients with good psychometric performance versus a structured clinical interview; categorical interpretations followed the published validation framework [15]. Evidence from the brief/French adaptation supports stability of the underlying dimensions across settings [14].

Clinical and prescribing data were abstracted from the EMR and prescription registry using a standardized form. Our demographic and clinical covariates were as follows:

- Sex (female/male), age (years), employment (paid employment vs. not), comorbidities (any documented medical comorbidity; coded as present/absent for analyses).
- Primary BZD indication (insomnia; anxiety; affective disorder; mixed anxious–depressive), as recorded in the EMR.
- Prescribed BZD agents included alprazolam, clonazepam, and midazolam, which were the only benzodiazepines available at the institution. In rare instances, some patients were prescribed lorazepam; however, these cases were excluded due to its low frequency of use (*n* = 3).
- Daily dose (mg/day), taken from the prescription (“mg per 24 h”).
- Duration of BZD use (months), calculated from start date to the index BDEPQ-MX visit.

The BDEPQ-MX yields a total score (continuous) and categorical classifications reflecting the instrument’s four dimensions: No dependence, Pleasurable effects, Perceived need, and Dependence. In our first exploratory analysis, we dichotomized the scores into No dependence (both patients that scored “No dependence” and “Pleasurable effects”) and Any dependence (both patients that scored “Perceived need” and “Dependence”). In our second analysis, we explored differences between the four categories.

For regression analyses, we defined a binary endpoint “any dependence” (Perceived need or Dependence) versus No dependence (“No dependence” and “Pleasurable effects”); the continuous BDEPQ-MX total served as the outcome for the general linear model.

This was the census of eligible patients in the period; no a priori sample size calculation was performed. Missingness for model covariates was low; we used complete-case analysis. Sparse categories for BZD agents were combined into others to avoid quasi-separation.

We summarized continuous variables as mean ± SD and categorical variables as *n* (%). Group comparisons used the following:

- χ^2^ tests (or Fisher’s exact when appropriate) for categorical variables;
- Student’s *t* test for two-group comparisons of continuous variables;
- One-way ANOVA for comparisons across > 2 groups, followed by Tukey’s post hoc tests when indicated.

Effect sizes were reported as Cramer’s V (χ^2^) and partial η^2^ (ANOVA). Two multivariable models were specified a priori:

1. Logistic regression with any dependence as the dependent variable and the following covariates: age, sex, employment, comorbidity (present/absent), duration of BZD use (months), daily dose (mg/day), and BZD drug. We present adjusted odds ratios (aORs) with 95% CIs.
2. General linear model (GLM) with BDEPQ-MX total score as the dependent variable and the same covariates; given the clinical interest, we explored interaction terms (sex × employment, sex × comorbidity, employment × comorbidity, and sex × employment × comorbidity). We report F statistics, *p* values, and partial η^2^.

Model assumptions were checked: normality/homoscedasticity of residuals for the GLM; linearity of the logit for continuous predictors, influential observations, and multicollinearity (variance inflation factor < 5) for the logistic model. Two-sided α = 0.05 defined statistical significance. Analyses were conducted in SPSS v26 (IBM Corp., Armonk, NY, USA) with cross-checks in Python v3.10, (Python Software Foundation, Beaverton, OR, USA) for reproducibility.

## 3. Results

**Figure 1:**
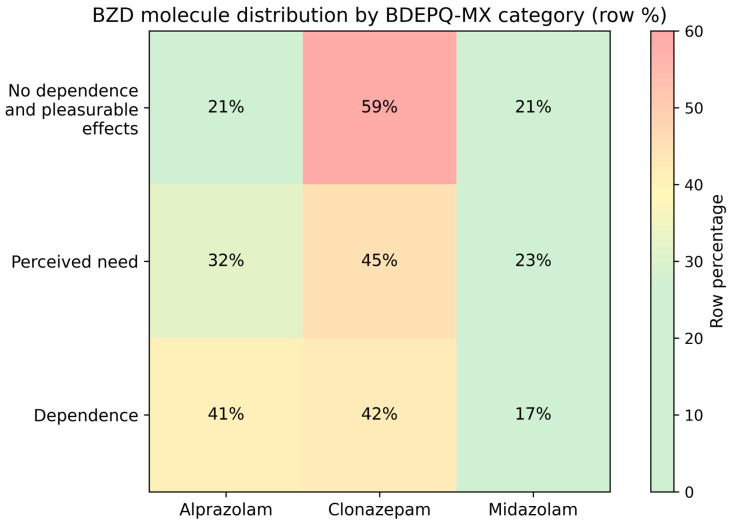
Heatmap of benzodiazepine drug by BDEPQ-MX category. Cells display the percentage of patients within each category receiving alprazolam, clonazepam, or midazolam; rows are normalized to 100% (minor rounding correction applied). Pastel green–yellow–red shading reflects lower-to-higher proportions. Categories: No dependence and Pleasurable effects (*n* = 73, both categories were unified due to the low sample in the pleasurable effects group), Perceived need (*n* = 31), Dependence (*n* = 77).

During the study period, 181 outpatients completed the BDEPQ-MX. The distribution of BDEPQ-MX categories is shown in Figure 1. Dependence was the most frequent category, followed by No dependence and Perceived need, with Pleasurable effects representing the smallest subgroup (Figure 1). For bivariate comparisons and multivariable models, complete-case analyses comprised 181 patients.

Compared with patients without any dependence, those with any BDEPQ-MX dependence were more often female and had a higher burden of comorbidities (both χ^2^, *p* < 0.001 and *p* = 0.006, respectively). Age, employment, and the distribution of clinical indications (insomnia, anxiety, affective, mixed anxious–depressive) did not differ significantly between groups (all *p* > 0.05). By contrast, duration of BZD use (months) showed a marked difference, with higher intake among those with any dependence (mean ± SD 22.6 ± 11.5 vs. 5.9 ± 4.9; *p* < 0.001). Daily dose (mg/day) did not differ between groups (*p* = 0.6). Regarding benzodiazepine medications, alprazolam was more frequent among patients with any dependence (38.9% vs. 20.5%; *p* = 0.009), whereas clonazepam was less frequent (43.5% vs. 58.9%; *p* ≈ 0.03); midazolam did not differ (*p* = 0.6) (Table 1).

### 3.1. Gradients Across BDEPQ-MX Categories

**Figure 2:**
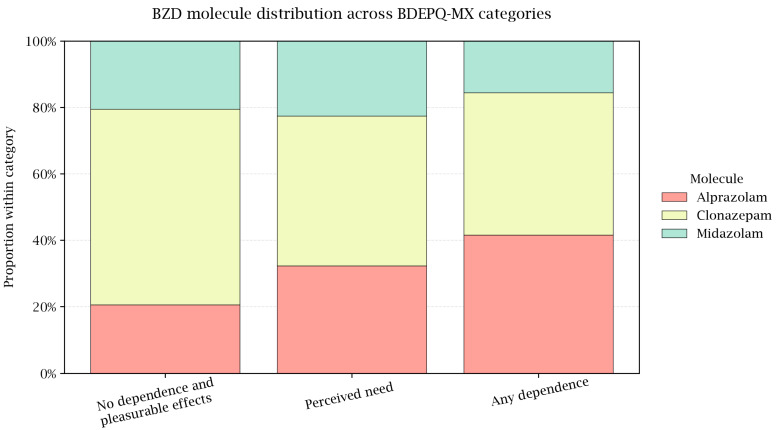
Benzodiazepine drug distribution across BDEPQ-MX categories (100% stacked bars). For each category—No dependence and Pleasurable effects (*n* = 73, both categories were unified due to the low sample in the pleasurable effects group), Perceived need (*n* = 31), Dependence (*n* = 77)—bars indicate the within-category proportion of alprazolam, clonazepam, and midazolam derived from Table 2 row percentages (normalized to 100% to address rounding). This plot visualizes the shift toward alprazolam in higher-dependence categories.

When the sample was stratified by ordered BDEPQ-MX categories, a clear exposure gradient emerged (Table 2). The proportion of women increased from the No dependence group and Pleasurable effects to Perceived need and Dependence (*p* < 0.001), and comorbidities became more prevalent across the same gradient (*p* = 0.015). Age, employment, and primary indication again showed no significant differences (all *p* > 0.05). The duration of BZD use (months) rose stepwise across categories (*p* < 0.001), whereas the daily dose did not vary (*p* = 0.7) (Table 2). Consistent with the two-group comparison, the drug mix shifted toward alprazolam in higher-dependence categories (*p* = 0.031), with a corresponding decrease in clonazepam (*p* ≈ 0.02), while midazolam remained similar (*p* = 0.5). These distributional patterns are visualized in row-percentage heatmap (Figure 2), which together show a higher share of alprazolam within Perceived need and Dependence and a lower share of clonazepam.

### 3.2. Multivariable Analyses

In the logistic regression for any dependence (Table 3), the male sex was independently associated with lower odds of dependence (aOR 0.29; 95% CI 0.11–0.76; *p* = 0.013), while the duration of BZD use (months) was strongly and positively associated with dependence (aOR 1.32 per month; 95% CI 1.20–1.45; *p* < 0.001). Age, employment, comorbidity, and daily dose were not significant predictors in the adjusted model (all *p* > 0.05).

In the general linear model with the continuous BDEPQ-MX total score as the outcome (Table 4), the duration of BZD use was again the dominant contributor (F = 203.26; *p* < 0.001; partial η^2^ = 0.545). No main effects were observed for age, daily dose, sex, employment, or comorbidity. Among interaction terms, only the sex × employment × comorbidity interaction reached statistical significance (F = 3.997; *p* = 0.047), with a small effect size (partial η^2^ = 0.023).

Overall, the results consistently indicate that longer exposure—rather than daily dose or nominal indication—is the key driver of BZP-related dependence phenomena in this outpatient pain and palliative-care population, with a sex effect favoring lower odds among men. Visualizations mirror the tabular contrasts, and multivariable models corroborate the central role of the duration of use.

## 4. Discussion

In our study, 59.7% of patients showed dependence on BZD agents with a mean use of 22.6 ± 11.5 months. Compared against the general population, BZD dependence levels are lower, between 6.6 and 6.8% [18]; however, in clinical settings where BZD agents are used to treat pain like our population, levels tend to be higher. Voyer P et al. screened BZD dependence levels in older adults in Quebec, observing a more similar prevalence to ours of 43% [19]. In another study performed in Spain in patients who reported using BZD for at least 1 month, De la Cuevas C. and Juan de la Fuente Emilio reported a dependence prevalence of 47% [20]. Only in one study, higher prevalence was reported: Kan C. et al. analyzed outpatient BZD users in the Netherlands, divided by users from the general practice and self-help patients; the first group have a prevalence of dependence of 40%, but in the second group, dependence levels reached up to 97% [21].

Factors associated with dependence we observed in our study were female sex, presence of chronic diseases, and length of usage (months). The female sex has been associated as a risk factor of dependence in other studies [20,22,23]. De la Cuevas C. and Juan de la Fuente Emilio also found that dependence was more prevalent in patients using BZD for more time [20]. Alongside Marriott S. and Tyrer P. also reported that longer therapies were risk factors associated with dependence [24]. An important risk factor commonly reported [25,26] that was not identified in our study was higher BZD dose; this could be due to the fact that all patients in our study were under a regime of a mean dose of 2 mg per day. It must be noted that emotional spectrum was not accounted for in our study as a risk factor, although it has been found to be a risk factor to BZD dependence by Kanopka A et al. which observed that introversion and adverse life events were risk factors [25], or the study performed by Ching-Fang Sun et al., where they observed that depressive disorder along with substance abuse were associated with BZD dependence [27].

The duration we observed in our study is similar to the broader literature. Systematic and cohort analyses identify persistence beyond 4–12 weeks as a key threshold after which dependence, tolerance, and withdrawal become increasingly likely, and a sizable fraction of new users transition to long-term use if treatment is not time-limited [28]. Current geriatric and safety guidelines, therefore, recommend restricting BZD courses to the short term, which can also be observed in our results, where shorter usage terms reflected less dependence prevalence [29].

Our drug-specific pattern—a higher share of alprazolam among dependence categories—has pharmacologic plausibility. Reviews note more severe and rapid-onset withdrawal with alprazolam than with many other BZDs, likely related to its short half-life/high potency and interdose withdrawal, which can reinforce continued use and dose-timing behaviors [30]. Notably, some cohorts link specific BZDs to transitioning into long-term use (e.g., nitrazepam, temazepam, lorazepam, clonazepam), underscoring that drug effects are context-dependent [31]. In our clinic, drug shifting was limited due to having only three BZD agents available at the pharmacy to prescribe.

Methodologically, strengths include the use of a validated instrument (BDEPQ-MX) to quantify dependence dimensions in a real-world population—in this case, patients with sleep and/or emotional disorders [15]—alongside the inclusion of a large number of patients using BZD for almost 2 years. Weaknesses in our study consider the lack of record regarding concomitant drugs used by the patient and low reliability of information due to use of EMR. Methodological limitations include the single-center nature of the study, the descriptive design, which does not allow causal inferences regarding BZD dependence, and the reliance on self-reported data obtained through the BDEPQ-MX questionnaire. Consequently, reporting bias may be present due to stigma, fear, or misunderstanding of the questionnaire items.

## 5. Conclusions

In this outpatient descriptive observational study conducted in a pain relief and palliative care institute, pharmacist-led BDEPQ-MX screening identified a substantial burden of benzodiazepine (BZD)-related dependence phenomena, with more than half of the evaluated patients meeting dependence criteria. The principal risk factor associated with dependence was the duration of BZD exposure, underscoring the importance of prioritizing treatment duration when assessing the dependence risk in clinical settings. The conduct of this study highlights an area for improvement within healthcare institutions, where the identification of conditions that place patients’ health at risk can be achieved through medical record review. Future research should focus on interventional and deprescribing programs for patients using benzodiazepines, led by pharmacists, thereby promoting the distribution of responsibilities within the multidisciplinary healthcare team.

## Figures and Tables

**Table 1:** Characteristics by any BDEPQ-MX dependence vs. No dependence.

Variable	No Dependence *n* = 73 (100.0)	Any Dependence *n* = 108 (100.0)	*p*
Female sex, *n* (%)	36 (49.3)	86 (79.6)	<0.001
Male sex, *n* (%)	37 (50.7)	22 (20.4)	
Age, mean ± SD (years)	56.3 ± 19.6	61.0 ± 18.4	0.1
Employment, *n* (%)	38 (52.1)	44 (40.7)	0.1
Comorbidities, *n* (%)	48 (65.8)	90 (83.3)	0.006
Insomnia, *n* (%)	21 (28.8)	37 (34.3)	0.4
Affective disorder, *n* (%)	19 (26.0)	23 (21.3)	0.4
Anxiety disorder, *n* (%)	19 (26.0)	33 (30.6)	0.5
Mixed anxiety–depressive disorder, *n* (%)	14 (19.2)	15 (13.9)	0.3
Duration (months), mean ± SD	5.9 ± 4.9	22.6 ± 11.5	<0.001
Daily dose, mean ± SD	1.9 ± 1.8	1.8 ± 1.7	0.6
Alprazolam, *n* (%)	15 (20.5)	42 (38.9)	0.009
Clonazepam, *n* (%)	43 (58.9)	47 (43.5)	0.042
Midazolam, *n* (%)	15 (20.5)	19 (17.6)	0.6

Quantitative variables are represented as means ± SD and analyzed by Student’s *t* test, while qualitative variables are represented by frequency and percentage; they were analyzed by a chi square test.

**Table 2:** Characteristics by BDEPQ-MX category.

Variable	No Dependence, *n* = 60 (100.0)	Pleasurable Effects, *n* = 13 (100.0)	Perceived Need, *n* = 31 (100.0)	Any Dependence, *n* = 77 (100.0)	*p*
Female sex, *n* (%)	31 (51.7)	5 (38.5)	24 (77.4)	62 (80.2)	<0.001
Male sex, *n* (%)	29 (48.3)	8 (61.5)	7 (22.6)	15 (19.5)	
Age, mean ± SD (years)	55.9 ± 19.9	58.4 ± 18.5	62.1 ± 21.5	60.5 ± 17.1	0.4
Employment, *n* (%)	32 (53.3)	6 (46.2)	15 (48.4)	29 (37.7)	0.3
Comorbidities, *n* (%)	38 (63.3)	10 (76.9)	23 (74.2)	67 (87.0)	0.0015
Insomnia, *n* (%)	17 (28.3)	4 (30.8)	9 (29.0)	28 (36.4)	0.7
Affective disorder, *n* (%)	17 (28.3)	2 (15.4)	6 (19.4)	17 (22.1)	0.6
Anxiety disorder, *n* (%)	14 (23.3)	5 (38.5)	12 (38.7)	21 (27.3)	0.3
Mixed anxiety–depressive disorder, *n* (%)	12 (20.0)	2 (15.4)	4 (12.9)	11 (14.3)	0.7
Duration (months), mean ± SD	4.4 ± 3.5	12.8 ± 4.6	11.4 ± 4.5	27.2 ± 10.4	<0.001
Daily dose, mean ± SD	1.9 ± 1.6	2.4 ± 2.7	2.0 ± 1.9	1.8 ± 1.6	0.6
Alprazolam, *n* (%)	14 (23.3)	1 (7.7)	10 (32.3)	32 (41.6)	0.031
Clonazepam, *n* (%)	35 (58.3)	8 (61.5)	14 (45.2)	33 (42.9)	0.2
Midazolam, *n* (%)	11 (18.3)	4 (30.8)	7 (22.6)	12 (15.6)	0.5

Quantitative variables are represented as means ± SD and analyzed by an ANOVA test, while qualitative variables are represented by frequency and percentage; they were analyzed by a chi square test.

**Table 3:** Risk of any BDEPQ-MX dependence (Dependence or Perceived need).

Variable	OR (Unadjusted)	95% CI	*p*	aOR (Adjusted)	95% CI	*p*
Daily dose (mg/day)	0.88	0.68–1.13	0.3	—	—	—
Age (years)	1.02	0.98–1.05	0.1	—	—	—
Male sex	0.30	0.11–0.81	0.018	0.29	0.11–0.76	0.013
Employment	1.36	0.41–4.43	0.6	—	—	—
Comorbidities	0.83	0.29–2.38	0.7	—	—	—
Duration (months)	1.34	1.21–1.48	<0.001	1.32	1.20–1.45	<0.001

Abbreviations: OR: odds ratio, CI: confidence interval, aOR: adjusted odd ratio, mg: milligrams.

**Table 4:** Linear regression model (ANOVA table for the GLM).

Source	Mean Square	F	*p*	Partial η^2^
Intercept	577.455	15.180	<0.001	0.082
Age	22.614	0.594	0.442	0.003
Daily dose	16.847	0.443	0.507	0.003
Duration (months)	7732.303	203.259	<0.001	0.545
Sex	40.206	1.057	0.305	0.006
Employment	2.265	0.060	0.808	0.000
Comorbidity	3.963	0.104	0.747	0.001
Sex × Employment	77.397	2.035	0.156	0.012
Sex × Comorbidity	64.704	1.701	0.194	0.010
Employment × Comorbidity	0.581	0.015	0.902	0.000
Sex × Employment × Comorbidity	152.049	3.997	0.047	0.023

Outcome is the BDEPQ-MX total score. F tests derive from the general linear model; *p* values reported as in the original table (values of 0.000 shown as <0.001). Partial η^2^ quantifies effect size.

## Data Availability

The data presented in this study are available on request from the corresponding author due to privacy and ethical restrictions on individual-level EMR data.

## Abbreviations

The following abbreviations were used in this manuscript:

ANOVA	Analysis of variance
aOR	Adjusted odds ratio
AUC	Area under the ROC curve
BDEPQ	Benzodiazepine Dependence Questionnaire
BDEPQ-MX	Mexican adaptation/validation of the Benzodiazepine Dependence Questionnaire
BZD	Benzodiazepine
CI	Confidence interval
EMR	Electronic medical record
GLM	General linear model
IRB	Institutional Review Board
OR	Odds ratio
SCID-I	Structured Clinical Interview for DSM-IV Axis I Disorders
SD	Standard deviation
SPSS	Statistical Package for the Social Sciences
VIF	Variance inflation factor
χ^2^	Chi-square test
η^2^ (partial η^2^)	(Partial) eta-squared, effect size
